# Assessing the vector competence of Italian *Culex pipiens* and *Aedes albopictus* mosquitoes for the re-emerging Oropouche virus

**DOI:** 10.1186/s13071-025-06912-x

**Published:** 2025-07-08

**Authors:** Elisa Mancuso, Francesco Severini, Luciano Toma, Giulia Marsili, Francesca Casale, Concetta Castilletti, Federico Giovanni Gobbi, Antonello Amendola, Christina Merakou, Martina Del Manso, Flavia Riccardo, Giulietta Venturi, Marco Di Luca, Claudia Fortuna

**Affiliations:** 1 Dipartimento Malattie Infettive, Istituto Superiore di Sanità, Rome, Italy; 2 Dipartimento di Malattie Infettive, Tropicali e Microbiologia, IRCCS Ospedale Sacro Cuore Don Calabria, Negrar di Valpolicella, Verona Italy; 3 Dipartimento di Science Cliniche e Sperimentali, Università di Brescia, Brescia, Italy

**Keywords:** OROV, Experimental infection, Virus transmission, Emerging arbovirus, Italy, Reassortant strain, Vector-borne disease

## Abstract

In 2024, Italy reported its first five cases of Oropouche fever in travelers returning from Cuba and Brazil. The Oropouche virus (OROV), an emerging *Orthobunyavirus* of the *Peribunyaviridae* family, is a zoonotic arbovirus responsible for febrile illnesses in humans, often misdiagnosed owing to its clinical similarity to those caused by dengue and Zika virus infections. Originally endemic to the Amazon region and first detected in Trinidad and Tobago in 1955, OROV has since spread throughout South and Central America. Recent outbreaks in Brazil and Cuba have been linked to the newly identified genotype V. Although *Culicoides paraensis* midges are recognized as the primary vector in the urban transmission cycle, their presence in Cuba was documented only after the outbreak. This late detection, coupled with a possibly low population density of this species, has raised concerns that other, more abundant arthropod species may have played a role in transmitting OROV to humans on the island. While some mosquito species have been implicated as potential OROV secondary vectors, their role remains uncertain. This study evaluates the vector competence of Italian populations of *Aedes albopictus* and *Culex pipiens* for the newly circulating OROV strain, which was introduced into the country by infected travelers. Experimental infections were conducted under biosafety level 3 conditions, exposing adult female mosquitoes to an infectious blood meal containing OROV. Viral presence was assessed using a real-time RT-PCR in mosquito bodies, legs + wings, saliva, and the first gonotrophic progeny (F1). Results indicated that, while both species successfully ingested infectious virus particles, *Cx. pipiens* exhibited no signs of infection, dissemination, or transmission at any time point. In *Ae. albopictus*, viral RNA was detected in two body samples but not in saliva or disseminated tissues, confirming an absence of transmission capability. In addition, no evidence of transmission to F1 generation was detected in either species. These findings are consistent with previous research on American mosquito populations, indicating that barriers to OROV transmission likely occur at the midgut level. Despite the apparent lack of vector competence in Italian *Ae. albopictus* and *Cx. pipiens*, the ongoing geographic expansion of OROV highlights the need for continued surveillance, particularly as the 2025 vector activity season approaches in Europe. The increasing circulation of the virus, along with its potential for adaptive evolution, highlights the importance of further research into the virus–vector interactions that could enable OROV to establish itself in new ecological niches.

**Graphical abstract:**

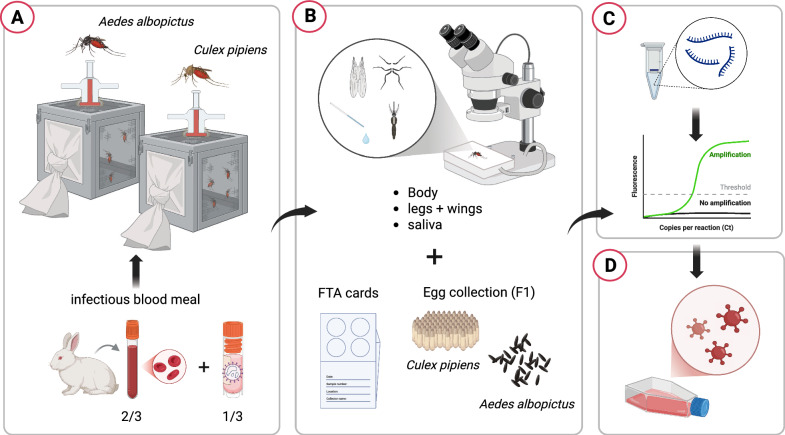

The Oropouche virus (OROV), classified under the genus *Orthobunyavirus* and the family *Peribunyaviridae*, is recognized as the causative agent of a zoonotic vector-borne disease that presents clinical symptoms very similar to those caused by dengue virus, Zika virus, or other febrile illnesses. Endemic to the Amazon region and first identified in Trinidad and Tobago in 1955, the virus has spread throughout the Caribbean and Central and South America over the years, with several reassortants [1], including the new strain, responsible for the recent outbreaks in Brazil and Cuba.

OROV exhibits a sylvatic cycle in forested regions, where vertebrate hosts, such as nonhuman primates, sloths, rodents, and birds, contribute to its circulation, alongside an urban epidemic cycle involving humans [2]. The virus primarily spreads to humans through the anthropophilic biting midge *Culicoides paraensis*, while in the sylvatic cycle, the primary arthropod vector remains unidentified [1]. However, mosquitoes such as *Culex quinquefasciatus*, *Coquillettidia venezuelensis*, and *Aedes serratus* have been found infected in natural settings [2–4].

The OROV genome consists of three single-stranded negative-sense RNA segments: small (S), medium (M), and large (L). The S segment encodes an overlapping open reading frame (ORF) for nucleocapsid and a nonstructural protein, the M segment encodes for two glycoproteins and a nonstructural protein, and the L segment encodes for an RNA-dependent RNA polymerase [5]. Like other multi-segmented viruses, OROV can reassort its genome segments. This mechanism, which occurs during genome replication after the coinfection of a single cell with multiple viruses, can generate progeny capable of altered virulence or immune evasion. Reassortment can also occur during coinfection with different OROV strains, favoring evolution and viral spread by altering vector competence or virulence [6, 7].

Public health concerns about OROV intensified in 2024 following an unprecedented increase in the incidence of human infections in Central and South America, including reports of four fatalities and cases of vertical transmission of the virus linked to miscarriages, fetal deaths, and microcephaly [8–10]. Contextually, the detection of OROV in human semen has raised questions regarding its potential for sexual transmission, emphasizing the need for further research in this area [11].

In the same year, the first 19 imported cases of Oropouche (ORO) fever were reported in EU countries [12], and Italy identified its first five cases in travelers returning from Cuba and Brazil [13]. To assess the risk of potential local transmission of OROV in temperate continental Europe, where known competent OROV vectors are not present, research on the competence of other local vectors is necessary to evaluate the current and potential future adaptation of OROV to new ecological niches.

Prior to 2024, vector competence studies focused on OROV were limited in number and primarily conducted on insect species that circulate in endemic regions or North America [2, 14, 15]. To date, no experimental studies have been carried out on European mosquito populations, leaving a significant gap in knowledge regarding the virus’s ability to establish itself in nonendemic regions. The objective of this study is to investigate, through controlled experimental infections, the potential vectorial role of Italian populations of *Aedes albopictus* and *Culex pipiens* in transmitting the newly circulating OROV strain introduced by infected travelers. This particular reassortant has been found to be genetically distinct from the four previously known OROV genotypes, clustering into a highly supported monophyletic clade. This newly identified genotype V also includes viral sequences associated with the 2022–2024 Brazilian outbreak [16].

The experimental work was conducted in a Biosafety Level 3 (BSL-3) facility using two mosquito colonies derived from field populations collected in Rome. *Culex pipiens* colony originated from larvae and *Ae. albopictus* from eggs collected using ovitraps employed for *Aedes* surveillance.

Both of the mosquito species were experimentally exposed to the first OROV strain isolated in Italy, obtained from a patient who had recently returned from Cuba in 2024 [16]. To initiate the infection process, adult female mosquitoes, aged 8–11 days, were allowed to feed for 1 h using a membrane feeder containing a mixture of rabbit blood and an OROV suspension. The final viral concentration of this suspension was 1.7 × 10^6^ TCID_50_/ml, and the temperature of the blood meal was maintained at 37 °C using a circulating warm water system. After feeding, only fully engorged females were transferred to a controlled climate chamber, maintained at a temperature of 26 ± 1 °C, 70% relative humidity, and a 14 h light/10 h dark photoperiod cycle. These mosquitoes were then sustained on a saturated sucrose solution and monitored for 21 consecutive days. For each mosquito species, a subset of five individuals was sampled at day 0 (immediately after blood feeding). In the case of *Ae. albopictus*, 20 mosquitoes were randomly collected at 7, 14, and 21 days post-exposure (dpe). For *Cx. pipiens*, 20 mosquitoes were sampled at 7 dpe, but due to high mortality rates, only 15 mosquitoes were available for collection at 14 dpe. At each collection time, mosquitoes were immobilized by placing them on a petri dish on ice and dissected by removing the legs and wings. Saliva was then collected by inducing salivation with the application of 1 mL of 1% pilocarpine solution to the body and placing the proboscis in a finely drawn quartz capillary tube filled with mineral oil (Fig. 1).

**Fig. 1:**
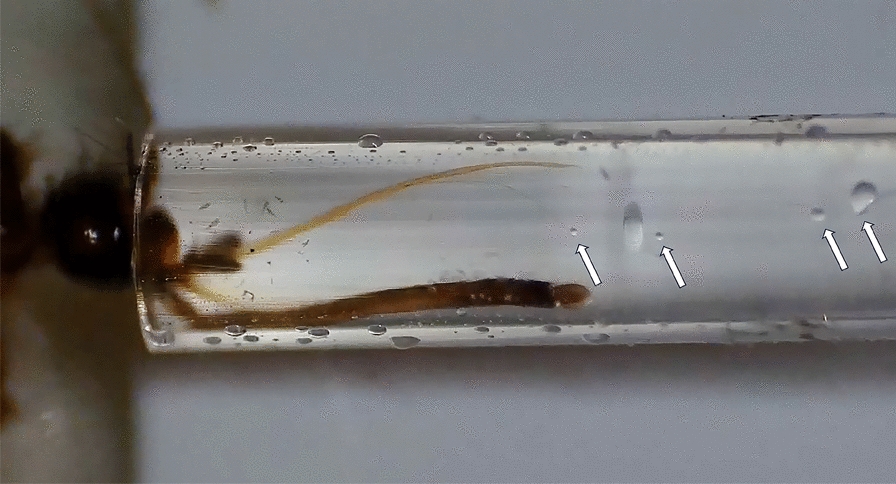
Detail of mosquito saliva collection following stimulation with pilocarpine 1% inside a capillary containing mineral oil. The arrows indicate the small drops of saliva that flow into a larger drop inside the oily medium

The bodies, legs + wings, and saliva of each mosquito were processed and analyzed separately to determine the presence of the OROV genome, allowing for the calculation of infection rate (IR), dissemination rate (DR), and transmission rate (TR) [17]. After the infectious blood meal, all engorged female mosquitoes of both species were allowed to lay eggs (first gonotrophic cycle, F1). The eggs were allowed to hatch, and the larvae developed into adulthood. For each species, ten pools of five adults, divided by sex (five male and five female pools), were tested for possible transmission of the virus to the F1 generation. The main phases of the experiment are schematized in Fig. 2.

**Fig. 2:**
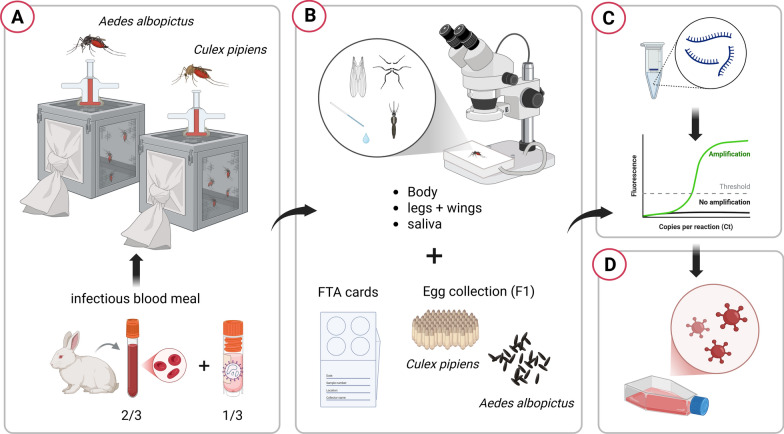
Main phases of the vector competence experiment: **A)** virus uptake via infectious blood meal and maintenance of engorged females; **B**) dissection of a selected number of individuals and collection of body, legs + wings, saliva, and Fast Technology for Analysis of Nucleic Acids (FTA) cards at 7, 14, and 21 days post-exposure. Eggs were collected throughout the experiment; **C**) RNA extraction from individual samples and molecular screening by real time RT-PCR; **D**) virus isolation from positive sample homogenates to check virus viability. Created in BioRender. Mancuso, E. (2025) https://BioRender.com/9ie8r8k

Fast Technology for Analysis of Nucleic Acids (FTA) cards were soaked in a sugar solution to collect mosquito saliva weekly and assess the potential presence of the virus throughout the experiment. The supernatant of samples that resulted in positive tests was filtered and inoculated onto Vero cell cultures to evaluate the viability of the virus. The development of cytopathic effects (CPE) in the cell cultures was used as a marker to confirm the presence of infectious virus particles.

The RNA was singularly extracted by each body, legs + wings, saliva, and FTA card specimens, and by pools of mosquitoes of the F1 generation by using the QIAsymphony DSP Virus/Pathogen Midi Kit in combination with the QIAsymphony SP (QIAGEN, Hilden, Germany). The OROV RNA presence in the different specimens was evaluated by the real Time RT-PCR protocol by RIVM Laboratory (National Institute for Public Health and the Environment, the Netherlands) modified from Weidmann et al. [18]. OROV quantification was obtained by comparing the crossing points of the values of the standard curve obtained from tenfold serial dilutions of OROV stocks, with estimated concentration by titration on Vero cells expressed as tissue culture infectious dose (TCID)_50_/ml.

All results described below are reported in Table 1. Specimens belonging to both *Cx. pipiens* and *Ae. albopictus*, which were collected immediately after being exposed to the OROV infectious blood meal (specifically at day 0), were tested and found to be positive for the presence of the virus. The mean viral titers measured in these individuals were 1.3 × 10^4^ TCID_50_/ml for *Cx. pipiens* and 1.4 × 10^4^ TCID_50_/ml for *Ae. albopictus*, thereby confirming that these mosquitoes had successfully ingested infectious virus particles during the blood meal. For *Cx. pipiens*, all tested specimens—including their bodies, legs + wings, saliva samples, and FTA cards tested negative for the presence of viral RNA at all collection time points. As a consequence, IR, DR, and TR were all determined to be zero, indicating a lack of infection, dissemination, and transmission potential for this species. In contrast, regarding *Ae. albopictus*, viral RNA was detected by real-time RT-PCR in one body sample at 7 dpe and in another body sample at 21 dpe, with cycle threshold values equivalent to viral titers of 1.7 × 10^5^ TCID_50_/ml and 1.15 × 10^6^ TCID_50_/ml, respectively. These results led to a cumulative IR of 3.3%, calculated as the number of infected mosquito bodies divided by the total tested. When the supernatant of the two OROV-positive body homogenates was inoculated onto Vero cells, viable virus was confirmed to be present. This was demonstrated by the appearance of CPE at times consistent with the viral titers found in the bodies: 6 days after inoculation for the “7 dpe-positive” body, and 4 days after inoculation for the “21 dpe-positive” body.

**Table 1:** Viral RNA determination in the tested specimens during the experiment

		Real time RT-PCR results				
	dpe	Body pos/*n* (%)	Legs + wings pos/*n* (%)	Saliva pos/*n* (%)	FTA card	F1 Generation (5 pools/sex)
*Culex pipiens*	T0	5/5 (100)	NA	NA	NA	neg
	T7	0/20 (0)	0/20 (0)	0/20 (0)	neg	
	T14	0/15 (0)	0/15 (0)	0/15 (0)	neg	
	T21	–	–	–	–	
*Aedes albopictus*	T0	5/5 (100)	NA	NA	NA	neg
	T7	1/20 (0,2)	0/20 (0)	0/20 (0)	neg	
	T14	0/20 (0)	0/20 (0)	0/20 (0)	neg	
	T21	1/20 (0,2)	0/20 (0)	0/20 (0)	neg	

*dpe*, days post-exposure

However, viral RNA was not detected in any of the legs + wings, saliva samples, or FTA cards of this species, indicating a complete absence of disseminated infection as well as an inability to transmit the virus.

Finally, for both mosquito species examined in this study, no virus particles were detected in the F1 generation. Nevertheless, since the results obtained from the first gonotrophic cycle may not be indicative of potential viral transmission to the eggs, further studies in this direction would be advisable to definitively rule out vertical transmission, especially if these populations were to become competent for OROV transmission in the future.

In recent years, the OROV has re-emerged as a significant public health concern, posing a growing threat to human populations in various regions. This resurgence has been characterized by an increasing frequency of epidemics, some of which have occurred for the first time in certain South American and Caribbean countries, such as Cuba. The notable rise in the number of reported cases within endemic areas, combined with the occurrence of cases imported by travelers returning from affected regions to previously unaffected areas, such as Europe and the United States, suggests a broader pattern of viral expansion and circulation [19]. Although scientific evidence strongly supports the idea that *C. paraensis* midges are more effective vectors of OROV than mosquitoes in the urban cycle [20], the lack of detection of this species in Cuba—where one of the most recent outbreaks occurred—initially suggested a potential role for mosquitoes in transmitting OROV to humans. However, their presence was finally documented for the first time in Cuba in March 2025, following extensive surveillance conducted after the outbreak. While all previously used traps proved ineffective, the midges were caught by human landing catch [21]. Although this new evidence suggests that *C. paraensis* was likely already present at the start of the outbreak, the lack of detection could be due to the inadequate surveillance system or their low population density. This reinforces the importance of investigating the role of mosquitoes in OROV transmission and whether they may contribute to the establishment of an endemic circulation on the Island of Cuba [22]. Therefore, evaluating the vector competence of different mosquito populations remains a critical factor in preventing the virus’s potential spread to new geographic areas. A thorough understanding of the intricate virus–vector interactions is essential, particularly as the transmission and circulation of the virus could be influenced by the pathogen’s capacity to adapt to different vector species. In line with previous findings regarding American populations of *Cx. pipiens* and *Ae. albopictus*, our study indicates a lack of vector competence for the most recently circulating OROV strain in their Italian counterparts. Specifically, our results are consistent with those reported by Payne et al. [15], who demonstrated that experimentally infected American mosquito species were not competent vectors for either historical or recent OROV strains, as evidenced by an IR of 2% and the absence of transmission. Earlier research has demonstrated that multiple mosquito species, including *Ae. albopictus* and *Cx. pipiens*, fail to acquire the virus through natural feeding on infected mice. However, experimental thoracic microinjection of the virus into these mosquitoes leads to significantly increased viral titers, enabling subsequent transmission to naive immunodeficient mice [15]. In light of previous observations, our findings suggest that the primary barriers to infection and transmission are likely located at the midgut level. This finding is consistent with the conclusions drawn by Gallichotte et al. in their comprehensive systematic review of pre-2024 studies [14], which emphasized the importance of midgut barriers in restricting virus acquisition and dissemination rather than attributing these limitations to a fundamental molecular incompatibility between the virus and its mosquito hosts. Although our study did not detect any OROV infection in *Cx. pipiens*, a single positive saliva sample recently documented by Payne et al. [15], highlights the necessity for ongoing intraspecific surveillance among *Cx. pipiens* populations. While we acknowledge the limitations of this pioneering study—including the absence of biological replicates, limited sample sizes due to BSL-3 constraints, and the assessment of transmission only to the F1 generation—we consider these preliminary results important as they provide an early indication to health authorities of a negligible risk of OROV circulation by the two main arbovirus vectors in Italy and Europe. Future studies with expanded sample sizes, replicated experiments, different mosquito populations, and evaluation of subsequent gonotrophic cycles will be essential to elucidate the vector competence of these species fully. In summary, despite the experimental limits, our study provides evidence that does not support vector competence in Italian *Cx. pipiens* and *Ae. albopictus* mosquitoes for the recently circulating OROV strain in Cuba. Nevertheless, the possibility of co-evolutionary processes and shifts in vector–virus interactions facilitating the adaptation of OROV to new epidemiological contexts and promoting its geographical expansion should not be underestimated. The introduction of OROV fever cases into Italy and other nonendemic regions in 2024, along with the increasing circulation of the virus throughout much of Central and South America, reinforces the importance of maintaining a high level of vigilance as the 2025 vector activity season in Europe approaches. Sustained vector surveillance efforts, combined with comprehensive research on potential transmission dynamics, will be crucial in mitigating the public health risks associated with the continued spread of this emerging arbovirus.

## Data Availability

Data supporting the main conclusions of this study are included in the manuscript.
